# Supplementary figures and images for: Baloxavir treatment of ferrets infected with influenza A(H1N1)pdm09 virus reduces onward transmission
Source: PLoS Pathog. 2020 Apr 15;16(4):e1008395. doi: 10.1371/journal.ppat.1008395 (PMC7159184; doi:10.1371/journal.ppat.1008395)

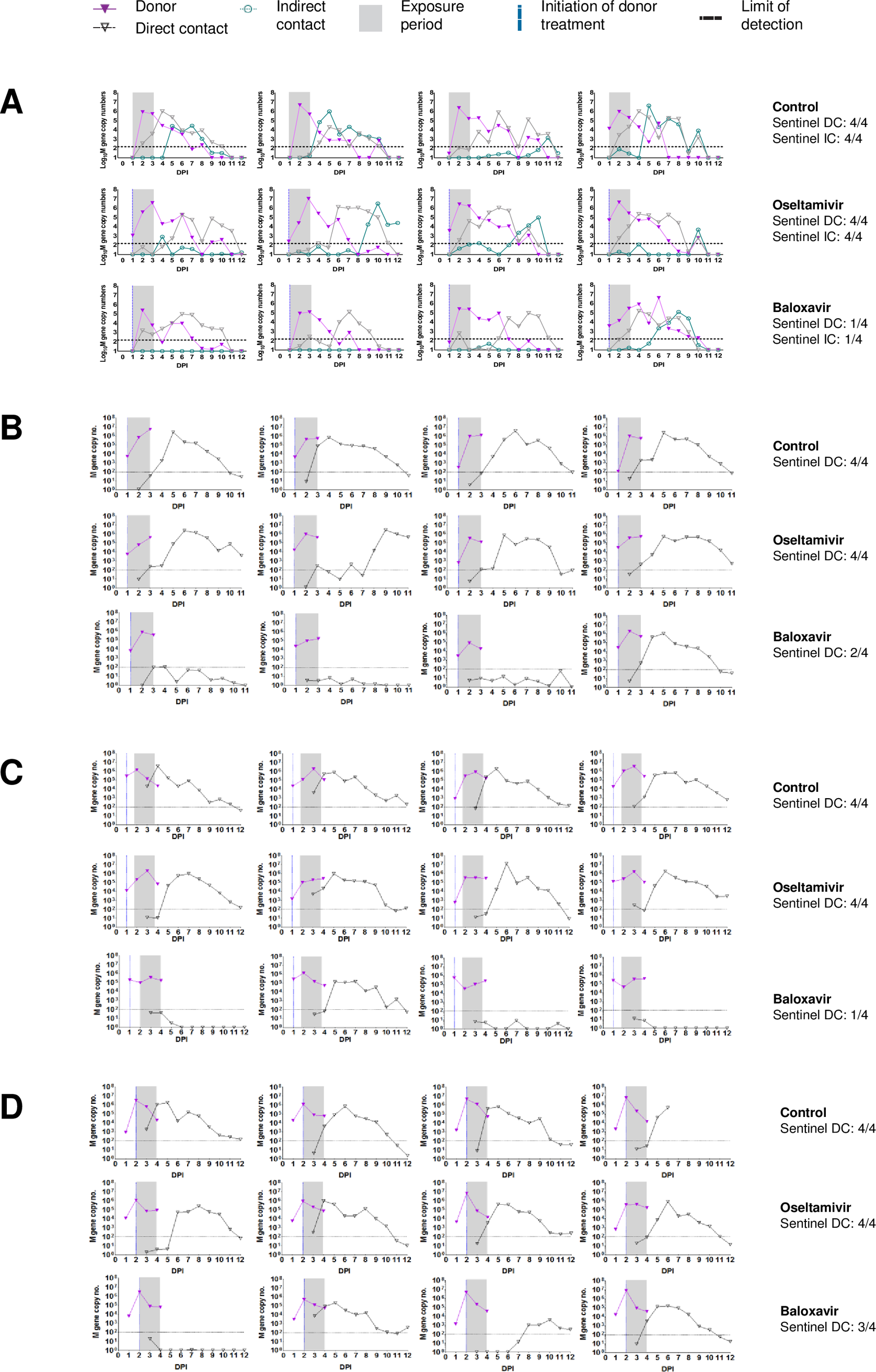

Supplement: S1 Fig — Copies of the influenza A M gene per μL RNA are displayed. London: Donor ferrets were intranasally inoculated with 104 PFU of influenza A/England/195/2009. Melbourne: Donor ferrets were inoculated with 103 TCID50 of influenza A/Perth/265/2009 by the intranasal route. (A) (London) donor ferrets receiving no treatment (upper panel), OST (middle) or BXA (lower) at 24 hours p.i. were immediately exposed to naïve DC and IC sentinels. Log-transformed graphs are shown. (B) (Melbourne) donors treated at 24 hours p.i. were immediately co-housed with naïve DC sentinels. (C) (Melbourne) donors treated with placebo (upper), OST (middle) or BXA (lower) at 24 hours p.i. were co-housed with naïve DC sentinels 24 hours later (D) (Melbourne) donors treated with antivirals at 48 hours p.i. were immediately co-housed with naïve DC sentinels. (TIF) [file ppat.1008395.s001.tif]
