# Supplementary material for: Baloxavir treatment of ferrets infected with influenza A(H1N1)pdm09 virus reduces onward transmission
Source: PLoS Pathog. 2020 Apr 15;16(4):e1008395. doi: 10.1371/journal.ppat.1008395 (PMC7159184; doi:10.1371/journal.ppat.1008395)
Supplement: S1 Table — (DOCX) [file ppat.1008395.s002.docx]

**S1 Table.** Detection of A(H1N1)pdm09 virus infection in each individual sentinel animal by viral culture, qRT-PCR and serum antibody response.

| 1. **(Lon) 24 hours p.i. donor treatment, immediate exposure (DC)** | | | | | | | | | | | | |
| --- | --- | --- | --- | --- | --- | --- | --- | --- | --- | --- | --- | --- |
| Sentinel positivity by indicated assay | Control | | | | OST | | | | BXA | | | |
| Virus titration (plaque)  [first positive DPI] | +  [4] | +  [3] | +  [5] | +  [5] | +  [6] | +  [6] | +  [5] | +  [5] | +  [5] | +  [5] | +  [7] | +  [5] |
| qRT-PCR  [first positive DPI] | +  [2] | +  [4] | +  [4] | +  [2] | +  [4] | +  [4] | +  [2] | +  [2] | +  [2] | +  [3] | +  [2] | +  [3] |
| HI on 14 DPI  [titre] | +  [640] | +  [1280] | +  [5120] | +  [2560] | +  [5120] | +  [20480] | +  [1280] | +  [2560] | +  [640] | +  [1280] | +  [5120] | +  [2560] |
| 1. **(Lon) 24 hours p.i. donor treatment, immediate exposure (IC)** | | | | | | | | | | | | |
| Sentinel positivity by indicated assay | Control | | | | OST | | | | BXA | | | |
| Virus titration (plaque)  [first positive DPI] | +  [4] | +  [5] | - | +  [5] | - | +  [10] | +  [9] | +  [9] | - | - | - | +  [8] |
| qRT-PCR  [first positive DPI] | +  [5] | +  [4] | +  [11] | +  [5] | - | +  [4] | +  [4] | +  [10] | - | - | - | +  [6] |
| HI on 14 DPI  [titre] | +  [640] | +  [1280] | -  [<10] | +  [2560] | -  [<10] | -  [<10] | +  [1280] | +  [2560] | - [<10] | -  [<10] | -  [<10] | +  [2560] |

| 1. **(Mel) 24 hours p.i. donor treatment, immediate exposure (DC)** | | | | | | | | | | | | |
| --- | --- | --- | --- | --- | --- | --- | --- | --- | --- | --- | --- | --- |
| Sentinel positivity by indicated assay | Control | | | | OST | | | | BXA | | | |
| Virus titration (TCID_50_)  [first positive DPI] | +  [4] | +  [3] | +  [4] | +  [5] | +  [5] | +  [8] | +  [5] | +  [4] | - | - | - | +  [3] |
| qRT-PCR  [first positive DPI] | +  [4] | +  [3] | +  [4] | +  [3] | +  [3] | +  [3] | +  [3] | +  [3] | +  [4] | - | - | +  [3] |
| HI  [not done] | N/A^#^ | | | | | | | | | | | |
| 1. **(Mel) 24 hours p.i. donor treatment, 24 hours delayed exposure (DC)** | | | | | | | | | | | | |
| Sentinel positivity by indicated assay | Control | | | | OST | | | | BXA | | | |
| Virus titration (TCID_50_)  [first positive DPI] | +  [3] | +  [4] | +  [4] | +  [4] | +  [5] | +  [4] | +  [5] | +  [5] | - | +  [5] | - | - |
| qRT-PCR  [first positive DPI] | +  [3] | +  [3] | +  [4] | +  [3] | +  [5] | +  [3] | +  [5] | +  [3] | - | +  [5] | - | - |
| HI on 18 DPI  [titre] | +  [5120] | +  [2560] | +  [5120] | +  [5120] | +  [5120] | +  [5120] | +  [2560] | +  [1280] | +  [40] | +  [2560] | -  [<20] | -  [<20] |
| 1. **(Mel) 48 hours p.i. donor treatment, immediate exposure (DC)** | | | | | | | | | | | | |
| Sentinel positivity by indicated assay | Control | | | | OST | | | | BXA | | | |
| Virus titration (TCID_50_)  [first positive DPI] | +  [3] | +  [4] | +  [4] | +  [5] | +  [6] | +  [4] | +  [4] | +  [5] | - | +  [3] | - | +  [4] |
| qRT-PCR  [first positive DPI] | +  [3] | +  [4] | +  [4] | +  [5] | +  [6] | +  [4] | +  [4] | +  [5] | - | +  [3] | +  [8] | +  [4] |
| HI on 18 DPI  [titre] | +  [10240] | +  [2560] | +  [5120] | N/A^*^ | +  [2560] | +  [10240] | +  [10240] | +  [5120] | +  [80] | +  [5120] | +  [320] | +  [2560] |

+ Ferret was influenza positive by the respective assay; - Ferret was influenza negative by the respective assay

^#^Serology samples were not collected for this experiment

*Ferret was sacrificed for ethical concerns at 4 DPE before development of a detectable antibody response
