# Supplementary material for: Baloxavir treatment of ferrets infected with influenza A(H1N1)pdm09 virus reduces onward transmission
Source: PLoS Pathog. 2020 Apr 15;16(4):e1008395. doi: 10.1371/journal.ppat.1008395 (PMC7159184; doi:10.1371/journal.ppat.1008395)
Supplement: Attachment — Submitted filename: Response to reviewers 3 Feb 2020.docx [file ppat.1008395.s003.docx]

***Authors responses are show in italics***

**Part I - Summary**
Please use this section to discuss strengths/weaknesses of study, novelty/significance, general execution and scholarship.

Reviewer #1: In this well-written article, authors present pharmacokinetic data from ferrets treated with baloxavir, and demonstrate that one treatment of BXA in influenza virus-inoculated ferrets reduces viral load in the upper respiratory tract and leads to a reduced frequency of virus transmission to contact animals compared with multi-day oseltamivir treatment, with no emergence of resistant viruses. The manuscript is notable in that these findings are demonstrated in two separate research groups, using two separate transmission models, challenge viruses, and experimental designs, supporting that these data are reproducible across different experimental conditions. Experiments are well-controlled, data is generally clearly presented, and results will be of interest to the field. **However, there are areas of the manuscript (notably with regard to harmonization between presentation of two different methodologies employed, and clarity in some presentation of results and experimental conditions) that would benefit from additional attention.**

*As indicated in detail below, the manuscript has now been modified to improve harmonization between the methodologies used in the two laboratories and in the clarity of results presentation.*

Reviewer #2: In 2018, the arsenal of antiviral treatment options for influenza was extended with an approval in the United States and Japan of Baloxavir marboxil (BXM), the influenza cap-dependent endonuclease inhibitor. One of the characteristic features of BXM is rapid reduction of influenza virus shedding in the upper respiratory tract of treated patients. However, the implication of rapid reduction of virus load in the upper respiratory tract on transmissibility was unknown. In this study the authors addressed the question whether BXM treatment can affect transmissibility to contacts of influenza A(H1N1)pdm09 virus in ferrets, the gold standard animals for evaluating transmission. Ferret transmission experiments were conducted in two independent laboratories: Imperial College London, London, UK and The Peter Doherty Institute for Infection and Immunity, Melbourne, Australia.

Reviewer #3: The manuscript submission by Wendy S. Barclay and colleagues reports on the efficacy of Baloxavir therapy to prevent transmission of influenza viruses among ferrets. A strength of the study is the independent assessment of Baloxavir therapy at two different research institutions. The authors report the pharmacokinetic (PK) profile of Baloxavir in ferrets. Treatment with Baloxavir reduced transmission among ferrets; however, Oseltamivir therapy was less efficacious in reducing transmission of influenza virus among ferrets. **There are two major weaknesses to the study. Ferrets were delivered the active form of Baloxavir, which is immediately active against influenza viruses; however, ferrets were given oral administration of Oseltamivir phosphate, which must be metabolized to the active form, oseltamivir carboxylate. The other weakness to the study is that the PK profile of Oseltamivir was not assessed following treatment of ferrets. Thus, there is not an equal comparison between the efficacy of Baloxavir versus Oseltamivir.**

*The concerns of Reviewer #3 regarding PK are addressed below in detail. In short, additional detail has now been provided to demonstrate that the active form of baloxavir is the appropriate choice for studies in ferrets, given that it most closely aligns with the PK profile seen in humans. To address the second point, additional detail has been added describing the previously published and extensive study evaluating the PK profile of oseltamivir in ferrets that informed the dose used in this study.*

**Part II – Major Issues: Key Experiments Required for Acceptance**
Please use this section to detail the key new experiments or modifications of existing experiments that should be absolutely required to validate study conclusions.

Generally, there should be no more than 3 such required experiments or major modifications for a "Major Revision" recommendation. If more than 3 experiments are necessary to validate the study conclusions, then you are encouraged to recommend "Reject".


Reviewer #1: (No Response)

Reviewer #2: 1. The experimental design and conditions of conducting experiments in a ferret model in two different laboratories were different. Importantly, the obtained results also differ in two different laboratories, as in London (where both DC and IC route of transmission were studied), the author did not see effect of BXA treatment on A(H1N1)pdm09 virus transmission to DC ferrets. In Melbourne (where only DC route of transmission was studied), the authors saw the effect of BXA treatment on A(H1N1)pdm09 virus transmission to DC ferrets. The authors discussed the factors affecting the obtained results. However, they must also include that in London recombinant influenza A(H1N1)pdm09 virus was used and in Melbourne – virus isolated from patient.

*This has now been added in the discussion such that the sentence now reads: “Other possible reasons include differences in the viruses (a reverse genetics derived recombinant strain was used in the London experiments whilst a cell culture isolate of a naturally circulating virus was used in the Melbourne experiments), ferrets and ambient conditions of the experiment (i.e. temperature and humidity) all of which may influence transmission dynamics (16, 35).”*

At the beginning of Results section, the authors should indicate criteria used to conclude about the virus transmissibility to DC and IC ferrets. For example, the following criteria were used: 1) AUC of infectious virus load over entire course of infection, 2) mean time to first virus-positive nasal wash; 3) frequency of transmission of virus. The same criteria must be used for analysis of the data in both laboratories.

*We have now included these criteria stating that: “Analyses to evaluate the effect of antiviral treatment of donor animals on viral shedding involved 1) area under the curve (AUC) analysis of plaque assay/TCID_50_ data and 2) viral titre comparisons at key time points.” and stating that “Analyses to evaluate the transmission of influenza virus from donor animals to sentinel animals involved 1) frequency of ferrets that became virus positive (by plaque assay/TCID50 or RT-PCR on any day) or HI serology positive, and 2) time (days) to first virus positivity.”*

*Some changes have been made to the results to ensure that the data for these criteria are described, as appropriate, for both laboratories.*

2. The data on clinical signs of infection was not presented and was not described. However, the authors were monitoring weight, temperature and activity of ferrets. Please provide a brief description of the obtained results.

*The clinical data was not particularly informative, and therefore for brevity was not included in the original manuscript. However, the authors agree that some inclusion of this data would be useful, in particular the presentation of clinical signs in the donor animals that either received an antiviral or placebo is most relevant. Brief comments on these aspects have now been included at the appropriate points in the results.*

3. The influenza virus titers in nasal washes of ferrets are presented in individual animals (n = 4/group, Figs. 2 - 5). It will be helpful for the readers if mean virus titers ± SD will be provided for each treatment group (control, OST, BXA) and statistical differences will be indicated.

*The use of mean virus titre measurements for a group of animals where only some animals are infected and others are not (i.e. in the sentinel ferrets) can be misleading and as such the authors are reluctant include such data. This sort of calculation is most appropriate for the donor animals in this study as all n=4 ferrets became infected. Currently we have reported the virology differences between the groups (control, OST, BXA) by presenting AUC +/- SD for the Melbourne experiments in the text and have now added in the data for the London experiments. The authors feel that an AUC calculation better captures the viral shedding profile of the ferrets than a mean viral titre and therefore would rather not add this additional calculation to the manuscript.*

4. Abstract must be modified. The current Abstract contains well-known information about influenza vaccines and antiviral drugs (half of the body of the Abstract) and very brief description of obtained results. PK data was not included. The Author summary must be modified – only two last sentences of summary are related to the current study.

*The reviewer raises an important point. Both the abstract and author summaries have been modified to include more outcomes that were derived from the study and less background information.*

Reviewer #3: The authors should address the PK profile of Oseltamivir in ferrets, especially for the test regimen described in the current study, which is not the standard of patient care. The authors should also revise statements pertaining to the enhanced therapeutic effect of Baloxavir over Oseltamivir since ferrets were treated with the active form of Baloxavir but the non-active form of Oseltamivir.

*For the results of an influenza antiviral animal model study to have relevance in informing human treatment it is important to conduct a pharmacokinetics analysis of in the animal being studied to see how the drug profile compares with that seen in humans who are receiving the approved dose concentration and frequency. It is common that the concentration of drug, the route of administration or the form of the drug (pro-drug vs active metabolite) may need to differ in the animal from that used in humans to achieve the same PK drug profile. See the following reference for further information:* ***Using the Ferret as an Animal Model for Investigating Influenza Antiviral Effectiveness****.* [*Oh DY*](http://bioinfo.bas.roche.com:8080/bicgi/pubmedgateway2?cmd=search&term=%22Oh%20DY%22%5BAuthor%5D)*,* [*Hurt AC*](http://bioinfo.bas.roche.com:8080/bicgi/pubmedgateway2?cmd=search&term=%22Hurt%20AC%22%5BAuthor%5D)*. Frontiers in microbiology. 2016; 7:80.*

*An extremely thorough and comprehensive oseltamivir PK analysis has already been completed in ferrets (Oseltamivir Population Pharmacokinetics in the Ferret: Model Application for Pharmacokinetic/Pharmacodynamic Study Design.* [*Reddy MB*](http://bioinfo.bas.roche.com:8080/bicgi/pubmedgateway2?cmd=search&term=%22Reddy%20MB%22%5BAuthor%5D)*,* [*Yang KH*](http://bioinfo.bas.roche.com:8080/bicgi/pubmedgateway2?cmd=search&term=%22Yang%20KH%22%5BAuthor%5D)*,* [*Rao G*](http://bioinfo.bas.roche.com:8080/bicgi/pubmedgateway2?cmd=search&term=%22Rao%20G%22%5BAuthor%5D)*,* [*Rayner CR*](http://bioinfo.bas.roche.com:8080/bicgi/pubmedgateway2?cmd=search&term=%22Rayner%20CR%22%5BAuthor%5D)*, [Nie J](http://bioinfo.bas.roche.com:8080/bicgi/pubmedgateway2?cmd=search&term=%22Nie%20J%22%5BAuthor%5D), [Pamulapati C](http://bioinfo.bas.roche.com:8080/bicgi/pubmedgateway2?cmd=search&term=%22Pamulapati%20C%22%5BAuthor%5D), [Marathe BM](http://bioinfo.bas.roche.com:8080/bicgi/pubmedgateway2?cmd=search&term=%22Marathe%20BM%22%5BAuthor%5D),* [*Forrest A*](http://bioinfo.bas.roche.com:8080/bicgi/pubmedgateway2?cmd=search&term=%22Forrest%20A%22%5BAuthor%5D)*, [Govorkova EA](http://bioinfo.bas.roche.com:8080/bicgi/pubmedgateway2?cmd=search&term=%22Govorkova%20EA%22%5BAuthor%5D). PloS one. 2015; 10(10):e0138069.). This study has been the basis for the selection of 5 mg/kg oseltamivir phosphate twice daily dosing in ferrets that has been used in this study and many others. It has been useful for the reviewer to highlight that we had not made reference to this study as background to the selection of the oseltamivir dose. We have now addressed this with additional text in both the introduction that reads: “A detailed pharmacokinetics (PK) analysis of OST in ferrets has been previously completed and a dose established that aligns to the typical PK profile of OST observed in humans following standard dosing” and in the methods that reads “The OST dose was based on a previous pharmacokinetic analysis that showed a 5 mg/kg dose of oseltamivir every 12 hours for 5 days results in the same median area under the plasma concentration-time curve drug concentration as observed in humans following the approved dose of 75 mg twice daily for 5 days”*

*The PK analysis of baloxavir described in this study found that subcutaneous administration of 4 mg/kg of the active form of baloxavir (BXA) was able to closely mimic the PK profile of BXA seen in humans following the approved oral dose of baloxavir marboxil. Therefore it was considered that this dosing would be more appropriate to inform human treatment questions than to simply deliver the drug at the same concentration and via the same route as it is given in humans.*

*Given that the dosing protocols of both baloxavir and oseltamivir in ferrets have been thoroughly evaluated to achieve PK levels that closely mimic human PK profiles the authors do not feel that it is necessary to revise statements pertaining to the conclusions regarding the effect of baloxavir or oseltamivir. Aspects related to drug PK in ferrets and the different formulations used are already addressed in the 2^nd^ paragraph of the discussion.*

**Part III – Minor Issues: Editorial and Data Presentation Modifications**
Please use this section for editorial suggestions as well as relatively minor modifications of existing data that would enhance clarity.


Reviewer #1: 1. The methods section states that administration of both BXM and BXA were evaluated in the ferret model (line 110), but methodology is only provided for BXA evaluation. Considering BXM data following oral delivery is presented in Figure 1, the authors should include additional information in the methods regarding BXM evaluation (dose/volume/diluent for oral evaluation).

*The authors apologise for mistakenly not including this in the original submission. This has now been addressed and additional text added to the methods section that now reads: “BXM and BXA were synthesized and provided by Shionogi & Co. Ltd. For oral BXM delivery, BXM was suspended in water containing 5% (w/v) sodium dodecyl sulfate (SDS) and 10% (w/w) polysorbate 80 (Wako Pure Chemical Industries, Osaka, Japan), and 10 mg/5 mL/kg delivered to fasted ferrets anesthetized by isoflurane using an intragastric tube.” Additional text on BXM administration has also been added to the Figure 1 legend.*

2. Interspersed throughout the methods and text, there are places where information is not uniformly described between London and Melbourne-based experiments, leading to potential confusion by readers regarding which properties are/are not similar or divergent. In example, line 154 (London) states that pre-inoculation sera was only tested against homologous virus whereas line 180 (Melbourne) states that pre-inoculation sera was screened against “recently circulating” influenza viruses; does this mean that London ferrets were not similarly screened? Similarly, Melbourne-based, but not London-based, experiments specify freezing ferret nasal samples prior to titration; does this imply that London samples were titered immediately post-collection without a freeze/thaw cycle?

*The authors have gone through the methods in detail and have now made a number of changes to address the non-uniformity between the method descriptions.*

3. The authors state that “IC and DC sentinels were exposed to donors (1:1) ratio” on line 171, but data presented in Figure 2 indicates one shared donor for both direct and indirect transmission experiments, so use of “1:1 ratio” appears incorrect or misleading here.

*To improve clarity the text has now been changed to read “IC and DC sentinels were exposed to the same donor ferret (1:1:1 ratio) for 48 hours.”*

4. A clearer depiction of duration of OST treatment needed in figure images/legends. Methods state that OST was delivered twice daily but it is unclear in panel A from figures 3 and 4 if OST treatment was ceased mid-contact with the donor ferret or not. It would be extremely helpful to the reader for the figure legend to include the total number of doses of OST doses received by inoculated ferrets.

*OST drug levels were administered twice daily to the donor ferrets such that drug was present during the entire period of contact with sentinel ferrets. As requested, and to improve clarity, additional text describing the number of OST doses received by donor ferrets has now been added to the figure 2-5 legends, as well as tablet symbols onto panel A of each of the figure to visually show the number of doses delivered.*

5. The discussion section is very informative and well-referenced with regard to the implications of these findings for humans, but it would still be of benefit for laboratorians to see this data presented in the context of other experiments conducted in ferrets. It would be helpful to include (even in just a limited capacity in places like lines 452-60 or 480-83) discussion of how these results compare to other in vivo challenge studies that have examined the ability of antiviral drugs to limit virus transmission to contact ferrets, so readers can understand the effect of BXA treatment relative to what has been shown previously (or not) in the literature with other intervention strategies.

*Additional text on this topic has been added to the discussion*.

6. The authors discuss on lines 490-1 differences in DC transmission efficiency between the London and Melbourne experiments and provide several potential explanations for this. It would be beneficial to include in the methods section the frequency of bedding change-outs in the two laboratory setups so the reader could better understand the potential for transmission in this system driven by virus-containing fomites in the cages.

*Additional text on this topic, stating that bedding litter was changed daily in both laboratories has been added to the Methods*.

7. Lines 155 and 160 in the methods are redundant regarding daily collection of body weights; one of these can be removed.

*This has now been corrected.*

8. Please specify in the methods volume and gauge needle employed to deliver BXA subcutaneously to ferrets.

*This has now been specified in the PK section of the methods*

9. It is unclear for the ‘placebo’ group in Figures 3-5 how this group was treated; did these ferrets receive sham sub-cutaneous injection (controlling for BXA delivery), sham sugar suspension orally (controlling for OST delivery), or both? This information (and how placebo differs from ‘control’ presented in Figure 2) should be more clearly articulated in the methods. Both ‘control’ and ‘placebo’ ferrets are described as ‘untreated’ in the figure legends so improved detail is needed.

*In the London experiments, control ferrets were untreated, whereas in the Melbourne experiments the control ferrets received placebo sham subcutaneous infection (controlling for BXA delivery).The text in the methods has now been modified to clarify: “In the Melbourne experiments, control animals received a single placebo dose of methyl cellulose solution (1 mL/kg) alone delivered subcutaneously in the same way as BXA, whereas in the London experiments control ferrets were untreated.” Modifications have also been made to the figure 3-5 legends to correctly replace “untreated” with “treated with placebo”.*

Reviewer #2: 1. Suggested change to title: “Baloxavir treatment of ferrets infected with influenza A(H1N1)pdm09 virus reduces onward transmission”.

*The requested change has been made*

2. Line 38 – word “sentinel” is not commonly used in the description of such kind of studies.

*The authors considered using the more typically used word “recipient”. However, this implies that a ferret will always be “receiving” viruses or an infection. The authors felt that the word “sentinel’ more accurately reflects their role in the study.*

3. Materials and methods – Include paragraph entitled “Compounds” and list all antiviral drugs used in the study, their source and method of preparation for PK and antiviral experiments.

*The requested change has been made*

4. Line 111 – Is it months or weeks? Please check.

*The authors confirm that months is correct.*

5. Line 118 - Spell out abbreviations LC-MS.

*The requested change has been made*

6. Line 138 – Here and throughout the text of the manuscript, change into “A/England/195/2009 (H1N1)pdm09”.

*The requested change has been made to the designations of both viruses used: A/England/195/2009 (H1N1)pdm09 and A/Perth/265/2009 (H1N1)pdm09*

7. Line 154 - provide reference for virus neutralization assay. Whether antibodies to other subtypes and influenza B virus were determined?

*The requested change has been made*

8. Line 167 - Indicated the distance between donors and IC animals.

*The requested change has been made*

9. Line 180 – Indicated erythrocytes used for HI assays.

*The requested change has been made*

10. Lines 210-216 - Indicate that a single dose of BXA was used, and twice daily OST for 5 days were used.

*The requested change has been made*

11. Line 303 – Explain why 6.85 ng/mL is the estimated target of effectiveness.

*The requested change has been made*

12. Supplementary Table S1 – Indicate in the notes to the Table the meaning “+” and “-“.

*The requested change has been made*

Reviewer #3: The experimental details for the ferret model are not completely described for all research sites. The PK studies were conducted at one site, but efficacy studies were conducted at two research institutions.

*As per the request from Reviewer #1 (suggestion #2) in this Part III, the authors have gone through the methods in detail and have now made a number of changes to address the non-uniformity between the method descriptions*

The oral gavage method for delivering Oseltamivir should be described in greater detail.

*Oseltamivir was not delivered by oral gavage, but instead by pipette to the mouth of ferrets. To improve clarity, additional detail has now been added to the “Antiviral treatment of ferrets” section of the Methods which now reads: “OST doses of 5 mg/kg were delivered twice daily (b.i.d.) (8h interval) for up to five days to non-sedated ferrets by providing it orally dropwise into the mouth of the ferret using a pipette (total dose 10 mg/kg/day).”*
